# Emergence and regional spread of extended-spectrum β-lactamase-producing *Klebsiella pneumoniae* ST307 at a Japanese tertiary-care hospital

**DOI:** 10.1128/spectrum.02526-25

**Published:** 2025-12-12

**Authors:** Wataru Ito, Ryuichi Nakano, Akiyo Nakano, Yuki Suzuki, Hisakazu Yano, Kei Kasahara

**Affiliations:** 1 Department of Infectious Diseases, Nara Medical University 12967, Kashihara, Nara, Japan; 2 Department of Microbiology and Infectious Diseases, Nara Medical University 12967, Kashihara, Nara, Japan; Universita degli Studi dell'Insubria, Varese, Italy

**Keywords:** *Klebsiella pneumoniae*, ST307, extended-spectrum β-lactamase, CTX-M-15, OXA-1, antimicrobial resistance

## Abstract

The global emergence of extended-spectrum β-lactamase-producing *Klebsiella pneumoniae* (ESBL-Kp), especially antimicrobial-resistant high-risk clones, is a public health concern. However, data on their molecular epidemiology in Japan remain limited. In this study, we conducted molecular analysis of 119 ESBL-Kp clinical isolates collected between January 2020 and April 2023 at a Japanese tertiary-care hospital. We identified 42 sequence types (STs), of which the most common was ST307 (*n* = 60, 50.4%), an antimicrobial-resistant high-risk clone. All ST307 isolates carried CTX-M-15, and 53 (88%) co-harbored OXA-1. Of the 60 ST307 isolates, 54 (90%) transferred IncFIIK plasmids carrying CTX-M-15 to *Escherichia coli* J53 recipients in conjugation experiments. ST307 isolates showed higher resistance rates than non-ST307 isolates, particularly to levofloxacin (100% vs 19%). Whole-genome sequencing of nine ST307 isolates revealed that *bla*_CTX-M-15_, *bla*_OXA-1_, and other antimicrobial-resistance genes were located on the same plasmid. All nine isolates had the GyrA S83I and ParC S80I substitutions. ST307 was found in both inpatients and outpatients, and core-SNP analysis revealed a narrow distance between inpatient and outpatient isolates, suggesting regional spread.

**IMPORTANCE:**

Sequence type (ST) 307 is an emerging antimicrobial-resistant high-risk clone of *Klebsiella pneumoniae* with global clinical relevance. This study is the first to document large-scale dissemination of ST307 in Japan, revealing its predominance among extended-spectrum β-lactamase-producing *K. pneumoniae* at a tertiary-care hospital. Detection in both inpatient and outpatient settings suggests community circulation. ST307 harbors multiple resistance genes on plasmids capable of spreading antimicrobial resistance between bacteria, with potential for interspecies transmission and limited treatment options. Comparative analysis of plasmid structures revealed high similarity to those reported globally, indicating possible international transmission routes. The identification of a conserved plasmid structure associated with ST307 may provide a useful genetic marker for tracking its spread. Continued molecular surveillance of ST307 and its plasmids is essential to understand the global epidemiology of its evolution and distribution and to inform infection control strategies.

## INTRODUCTION

*Klebsiella pneumoniae*, one of the Enterobacterales, has been traditionally associated with nosocomial infections such as urinary tract infections, pneumonia, and bacteremia (1). In recent years, concern has grown over the rise of multidrug-resistant *K. pneumoniae* strains that have acquired antimicrobial resistance (AMR). The World Health Organization has listed carbapenem- and third-generation cephalosporin-resistant *K. pneumoniae* as bacterial priority pathogens (2).

For *K. pneumoniae*, sequence types (STs) 11, 14, 15, 37, 101, 147, 258, 307, and their variants have been identified as AMR high-risk clones (3). A high proportion of these clones produces extended-spectrum β-lactamase (ESBL) or carbapenemase. However, their geographical distribution and the characteristics of other acquired resistance genes vary between countries and regions. Therefore, surveillance is being conducted in many countries to determine the spread of AMR and regional differences. However, regional surveillance is lacking in Japan, and the distribution of AMR high-risk clones remains unclear. Understanding regional AMR patterns is crucial for healthcare institutions to implement effective infection control strategies. Therefore, information on the regional epidemiological landscape of AMR high-risk clones in Japan is urgently needed.

In Japan, large-scale dissemination of the AMR high-risk clones of *K. pneumoniae* has not been reported to date. Our previous study showed that CTX-M-15 was the predominant ESBL type among ESBL-producing *K. pneumoniae* (ESBL-Kp) isolates and was detected across various ST clones (4). Although AMR high-risk clones such as ST15, ST101, and ST307 were detected in that study, their isolation frequency was low. Furthermore, another study on ESBL-Kp and our previous study on carbapenemase-producing *K. pneumoniae* also showed a low isolation frequency of AMR high-risk clones of global concern (5, 6).

However, recent genomic surveillance has suggested an increasing prevalence of ST307, highlighting the potential for localized spread of this high-risk clone (7). As ST307 is increasing in prevalence, replacing traditional clones such as ST258, it is considered a novel AMR high-risk clone of global concern (8). It was first recorded in the multilocus sequence typing (MLST) database from the Netherlands in 2008. Clinical isolates were first reported from the USA and Pakistan in 2013 (9, 10). It has subsequently been reported in various countries around the world. Furthermore, in some countries, the spread of ST307 clones that have acquired genes encoding carbapenemases, such as OXA-48, KPC, and NDM-1, has become a problem (11–13). Despite these findings, detailed data on regional distribution and clonal characteristics in Japan are lacking, highlighting the need for further epidemiological investigations.

This study aimed to elucidate the molecular epidemiological characteristics of ESBL-Kp in a Japanese tertiary-care hospital, focusing on the prevalence and dissemination patterns of AMR high-risk clones.

## MATERIALS AND METHODS

### Bacterial isolates

The study was conducted at Nara Medical University Hospital, a 992-bed tertiary university hospital in Japan. *K. pneumoniae* clinical isolates identified between January 2020 and April 2023 were investigated. Each isolate was identified with matrix-assisted laser desorption ionization time-of-flight mass spectrometry (MALDI-TOF MS) using a VITEK MS system v.3.2 (bioMérieux, Marcy-l’Étoile, France). If isolates were repeatedly identified from a single patient during the study period, only the first isolate from each patient was included in the analysis, regardless of the specimen type or whether it was a causative pathogen or a colonizing bacterium.

### Antimicrobial susceptibility testing and detection of carbapenemase production

ESBL production of *K. pneumoniae* was screened using CHROMagar ESBL (Kanto Kagaku, Tokyo, Japan). Isolates that grew on this medium were tested using the MAST D68C ESBL and AmpC detection set (Master Group, London, UK) to identify ESBL production. In addition, *K. pneumoniae* strains that did not grow on CHROMagar ESBL but were identified as suspected ESBL producers by the VITEK 2 Advanced Expert System (bioMérieux, Marcy-l’Étoile, France) were examined using the MAST D68C ESBL and AmpC detection set to ensure that no ESBL-Kp isolates were missed. All tests were performed once.

The antimicrobial susceptibility to various antimicrobial agents was determined as a single set using the agar dilution method according to the Clinical and Laboratory Standards Institute (CLSI) guidelines, and quality control was performed with *E. coli* ATCC 25922 (14). MICs were interpreted according to the breakpoints defined by the CLSI (15). The presence of carbapenemase was detected using the modified carbapenem inactivation method (mCIM), performed according to the CLSI guidelines (15). In order to focus on non-carbapenemase ESBL producers, mCIM-positive ESBL-Kp isolates were excluded.

### MLST

MLST was performed using seven housekeeping genes (*gapA*, *infB*, *mdh*, *pgi*, *phoE*, *rpoB*, and *tonB*) according to the method of Diancourt et al*.* (16). DNA sequence variations were analyzed by using an MLST database for *K. pneumoniae* (https://pubmlst.org/mlst). Novel STs were submitted to the curator (https://bigsdb.pasteur.fr/klebsiella/) and assigned new designations.

### PCR detection and DNA sequencing of β-lactamase genes

PCR was performed to detect β-lactamase genes, including *bla*_CTX-M-1 group_, *bla*_CTX-M-2 group_, *bla*_CTX-M-9 group_, *bla*_CTX-M-8/25 group_, *bla*_TEM_, *bla*_SHV_, and *bla*_OXA_ using AmpliTaq Gold 360 Master Mix (Thermo Fisher Scientific Inc., Japan) (17). Amplified PCR products of β-lactamase genes were sequenced on an Applied Biosystems 3730 DNA analyzer (Applied Biosystems, Carlsbad, CA, USA).

### Plasmid transferability and the replicon type

Transferability of the ESBL genes was studied by conjugation experiments with ESBL-Kp isolates as the donor and sodium azide-resistant *Escherichia coli* J53 as the recipient. The experiments were conducted using the broth-mating method, as described previously (18). The ESBL genes successfully transferred from the donor isolates were verified using PCR. The plasmid content of transconjugants was studied using PCR-based replicon typing (19, 20).

### Whole-genome sequencing (WGS)

WGS was performed on nine representative ST307 isolates selected to cover diverse OXA-1 status, isolation dates, specimen types, and inpatient/outpatient settings. Genomic DNA of the isolates was extracted using magLEAD 6gC (Precision System Science, Chiba, Japan) and sequenced using NovaSeq 6000 System (Illumina Inc., San Diego, CA, USA) and MinION (Oxford Nanopore Technologies, Oxford, UK). After read trimming and quality filtering, hybrid *de novo* assembly was performed using Unicycler v.0.5.0 (21). The assembled sequences were annotated using DFAST v.1.6.0 with standard settings (22). Acquired-resistance genes and chromosomal mutations were identified on the Center for Genomic Epidemiology server (http://www.genomicepidemiology.org/) using ResFinder v.4.6.0 (23). Comparison of the plasmid sequences was performed using BLASTn v.2.17.0 (https://blast.ncbi.nlm.nih.gov/Blast.cgi) and visualized using Easyfig v.2.2.2 (24).

### Chromosomal core single-nucleotide polymorphism (core-SNP) analysis

Core-SNP analysis was performed to assess the genetic relatedness of the nine ST307 isolates subjected to WGS. For this purpose, only chromosomal sequences were used, and plasmid sequences were excluded. Sequence reads were mapped to the *K. pneumoniae* HS11286 chromosome (GenBank accession no. CP003200) as the reference using the CLC Genomics Workbench v.24.0.2 (QIAGEN GmbH, Hilden, Germany) with default mapping parameters. To ensure accuracy, SNPs were called under the following thresholds: minimum coverage of 10×, minimum variant frequency of 90%*,* and minimum base quality score of 30. Putative SNPs located in repetitive or low-complexity regions were excluded, and only high-confidence SNPs in conserved chromosomal regions were retained for downstream analyses. Based on a previous study (25), isolates differing by fewer than 15 core-SNPs were considered clonally identical, whereas larger distances (e.g., >35 core-SNPs) were interpreted cautiously as indicative of a more distant relationship.

### Detection of capsular genotype, virulence genes, and string test for hypermucoviscous phenotype

Multiplex PCR was performed targeting the capsular polymerase genes *wzy_K1_* and *wzy_K2_*, and the virulence genes *rmpA*, *rmpA2*, *iroN*, and *iutA,* as described previously (26). In this study, *K. pneumoniae* isolates carrying two or more of these virulence genes were defined as potentially hypervirulent *K. pneumoniae* (4). The hypermucoviscous phenotype was identified using the string test, as described previously (27). The formation of a viscous string >5 mm long was defined as a positive result (27).

### Statistical analysis

Descriptive statistics were used to summarize the data, including AMR rates and other categorical or numerical variables. No inferential statistical analyses were performed, as the analyses were intended to provide a descriptive overview rather than hypothesis testing.

## RESULTS

### Bacterial isolates

Of the 1,074 *K*. *pneumoniae* clinical isolates identified during the study period, 136 were ESBL-Kp. The proportion of ESBL-Kp among *K. pneumoniae* isolates was 8.5% (30/352) in 2020, 10.4% (31/298) in 2021, 19.4% (63/325) in 2022, and 12.1% (12/99) in 2023. Of the 136 ESBL-Kp isolates, 121 were tested for mCIM, and 15 were excluded because no leftover samples were available in the hospital laboratory for testing. Of the 121 isolates tested for mCIM, two mCIM-positive isolates were excluded in order to focus on non-carbapenemase ESBL producers, leaving 119 mCIM-negative ESBL-Kp isolates in the study. These were isolated from urine (*n* = 36), sputum (*n* = 28), blood (*n* = 27), abscess (*n* = 14), stool (*n* = 6), bile (*n* = 3), bronchoalveolar lavage fluid (*n* = 1), gastric fluid (*n* = 1), ascites (*n* = 1), uterine (*n* = 1), and vaginal (*n* = 1) samples. Of the 119 ESBL-Kp isolates, 65 (54.6%) were isolated from patients more than 48 h after their admission (nosocomial isolates), and 54 (45.4%) were from outpatients or isolated from patients within 48 h after their admission (community-acquired isolates) (Supplemental Material; Table S1).

### MLST

MLST analysis identified 42 different STs among the 119 ESBL-Kp isolates, including one novel ST (ST7647) (Table 1). ST307 (*n* = 60) was the most frequently represented ST, followed by ST290 (*n* = 7), ST25, 29, 37, and 327 (*n* = 3 each), and ST14, 45, 54, and 86 (*n* = 2 each). The remaining 32 STs were each represented by one isolate. Among the AMR high-risk clones of global concern, ST14, 15, 101, and 307 were isolated, but ST11, 147, and 258 were not. The proportion of ST307 clones among ESBL-Kp isolates was 35.3% (6/17) in 2020, 31.0% (9/29) in 2021, 63.9% (39/61) in 2022, and 50.0% (6/12) in 2023. ST307 was isolated from urine (*n* = 21), blood (*n* = 16), sputum (*n* = 12), abscess (*n* = 5), stool (*n* = 3), bronchoalveolar lavage fluid (*n* = 1), gastric fluid (*n* = 1), and uterine (*n* = 1) samples. Of the 60 isolates of ST307, 38 (63%) were isolated from patients more than 48 h after their admission, and 22 (37%) were from outpatients or isolated from patients within 48 h after their admission (Supplemental Material; Table S1).

**TABLE 1:** Distribution of ESBL types and STs in 119 ESBL-producing *Klebsiella pneumoniae* isolates, and plasmid replicon types of the transconjugants^*a*^

ESBL type^*b*^	No. of isolates	ST^*c*^													No. of transferable isolates	Replicon types of transconjugants					Avg transfer frequency
		14	15	25	29	37	45	54	86	101	290	307	327	Others (30 STs)		FIIk	N	F	FIIk, A/C	Non-typable	
CTX-M-15 (1)	90	2	1	3	1		2	2		1		60	3	15^*d*^	68	57	1		7	3	1.2 × 10^−5^
CTX-M-3 (1)	2													2^*e*^	1				1		1.1 × 10^−5^
CTX-M-2 (2)	3								2					1 (ST36)	2		2				3.5 × 10^−5^
CTX-M-14 (9)	21					3					7			11^*f*^	15	10	2	2		1	4.0 × 10^−4^
CTX-M-27 (9)	1													1 (ST334)	0						NT
SHV-2	2				2										0						NT

^*a*^ ESBL, extended-spectrum β-lactamase; NT, not tested; ST, sequence type.

^*b*^ The numbers in parentheses represent CTX-M groups.

^*c*^ Underlined numbers represent antimicrobial-resistance high-risk STs.

^*d*^ Including STs 105, 191, 219, 392, 397, 477, 551, 716, 950, 2059, 2676, 2806, 3293, 5354, and 7647.

^*e*^ ST17 and ST1809.

^*f*^ Including STs 7, 23, 65, 70, 107, 268, 628, 1078, 2264, 3370, and 4857.

### Characterization of β-lactamases

Among 119 ESBL-Kp isolates, 117 were positive for CTX-M, and two were positive for SHV (Table 1). Among CTX-M positive isolates, 92 were from the CTX-M-1 group, 22 were from the CTX-M-9 group, and 3 were from the CTX-M-2 group. DNA sequencing revealed that the predominant type was CTX-M-15 (*n* = 90), followed by CTX-M-14 (*n* = 21). The CTX-M-15 group comprised 24 different STs. Most of these STs were represented by one to three isolates, except for ST307 (*n* = 60). All ST307 isolates produced CTX-M-15. The ESBL type of the two SHV-positive isolates was SHV-2.

Among the 119 ESBL-Kp isolates, 63 (52.9%) were positive for OXA, and DNA sequencing revealed that all OXA types were OXA-1. Of the ESBL-Kp isolates, 88% (53/60) of ST307 isolates and 17% (10/59) of non-ST307 isolates co-harbored OXA-1.

### Transferability of ESBL genes and plasmid replicon typing

The conjugation experiments showed that 86 of the 119 ESBL-Kp isolates (72.3%) were able to transfer their ESBL-encoded plasmids to *E. coli* J53 with an average transfer frequency of 9.8 × 10^−5^ (range 1.2 × 10^−8^ to 3.4 × 10^−3^) (Table 1). The most common plasmid identified among the 86 transconjugants had IncFIIK replicon (*n* = 75). All 60 ST307 isolates carried the IncFIB replicon, and 59 co-harbored IncFIIK replicon, of which 54 were IncFIIK plasmids transferred to *E. coli* J53. No IncFIB replicons were detected in the transconjugants. Four of 86 transconjugants were devoid of replicons (non-typable) for the incompatibility groups targeted by the PCR-based replicon typing method.

### Antimicrobial susceptibility profile

The results of antimicrobial susceptibility testing are shown in Table 2. AMR rates, expressed as percentages, between ST307 and non-ST307 isolates were compared descriptively. All the isolates showed resistance to cefotaxime and were significantly inhibited by clavulanate. For β-lactam antibiotics, the resistance rates to piperacillin-tazobactam (TZP), ceftazidime, cefepime, and aztreonam were higher in the ST307 isolates than in the non-ST307 isolates (17% vs 12%, 93% vs 31%, 68% vs 20%, and 98% vs 49%, respectively). For non-β-lactam antibiotics, levofloxacin (LVX), fosfomycin, gentamicin, and amikacin resistance rates were higher in the ST307 isolates than in the non-ST307 isolates (100% vs 19%, 100% vs 95%, 52% vs 36%, and 15% vs 0%, respectively).

**TABLE 2:** Susceptibility profile of ESBL-producing *Klebsiella pneumoniae* isolates^*a*^

Antimicrobial agents	ST307 (*n* = 60)				non-ST307 (*n* = 59)			
	Resistant (%)	MIC_50_ (μg/mL)	MIC_90_ (μg/mL)	MIC range (μg/mL)	Resistant (%)	MIC_50_ (μg/mL)	MIC_90_ (μg/mL)	MIC range (μg/mL)
Ampicillin	100	>256	>256	>256	100	100	>256	>256
Piperacillin	100	>256	>256	256–>256	100	>256	>256	64–>256
Piperacillin-tazobactam	17	8	128	2–>256	12	4	32	1–128
Cefmetazole	2	1	2	0.5–128	2	1	2	0.5–8
Cefotaxime	100	256	>256	64–>256	100	128	>256	4–>256
Cefotaxime-clavulanate	–^*b*^	≤0.06	≤0.06	≤0.06–0.5	–^*b*^	≤0.06	≤0.06	≤0.06–8
Ceftazidime	93	32	32	4–128	31	4	32	0.25–128
Cefepime	68	16	32	2–>256	20	8	16	0.5–64
Meropenem	0	≤0.06	≤0.06	≤0.06–2	0	≤0.06	≤0.06	≤0.06
Aztreonam	98	32	64	4–256	49	8	64	0.25–128
Levofloxacin	100	16	64	2–256	19	1	4	≤0.06–16
Fosfomycin	100	>256	>256	256–>256	95	>256	>256	128–>256
Gentamicin	52	32	64	0.25–128	36	0.5	128	0.25–128
Amikacin	15	8	16	1–16	0	4	8	1–8
Colistin	0	1	1	1–2	2	1	2	0.5–32

^*a*^ ESBL, extended-spectrum β-lactamase; ST, sequence type.

^*b*^ “–” represents that no resistance rate could be determined due to the absence of an established breakpoint.

### Genetic structures of the CTX-M-15-encoding plasmids

WGS was performed on the nine ST307 isolates, consisting of five inpatient isolates and four outpatient isolates, using next-generation sequencers to investigate the profiles of AMR genes other than *bla*_CTX-M-15_ and *bla*_OXA-1_.

**Fig 1:**
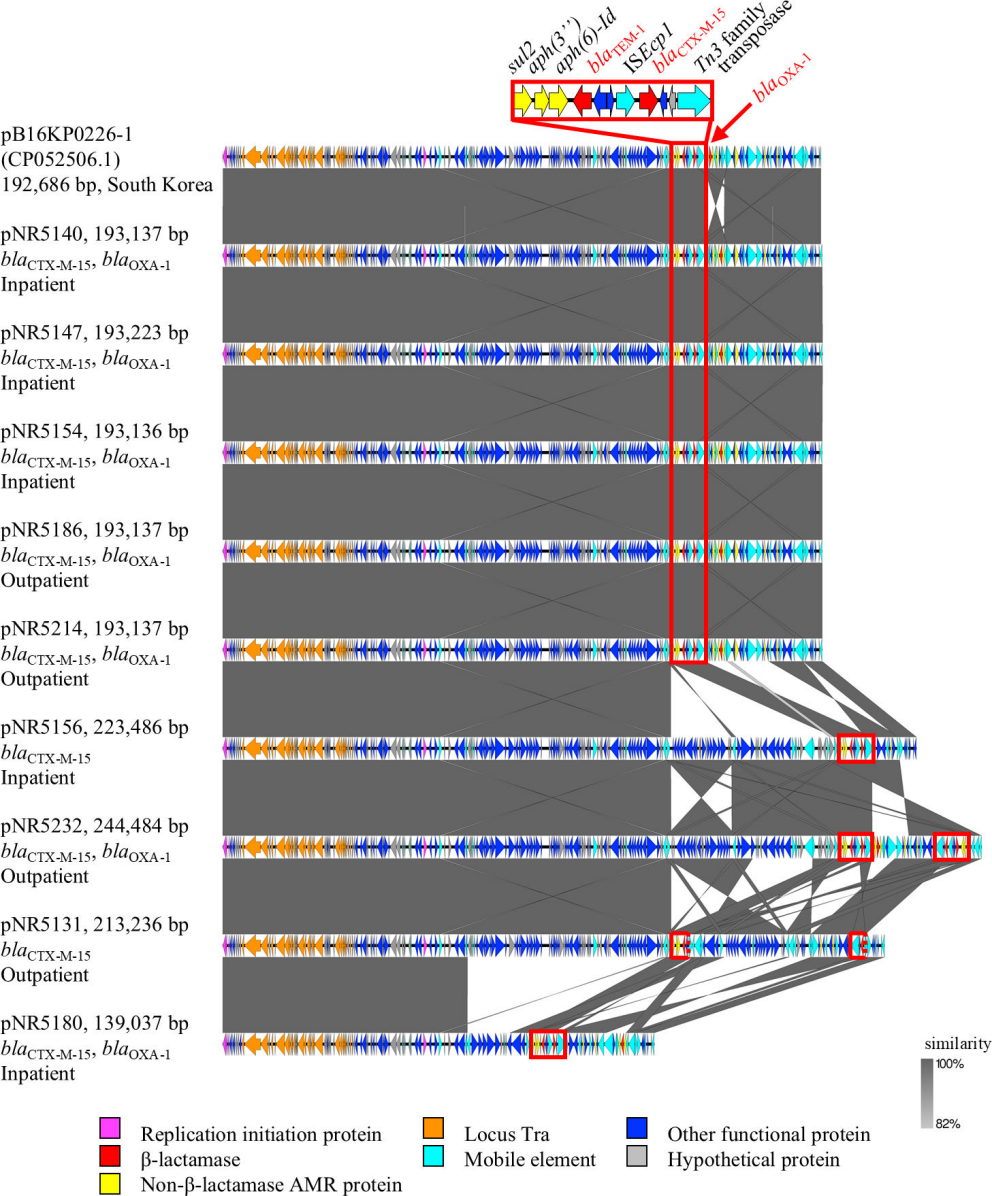
The genetic structures of the CTX-M-15-encoding plasmids isolated from nine *Klebsiella pneumoniae* ST307 isolates and a similar plasmid from the GenBank database. They share high similarity in the AMR gene cassette (highlighted in red), including *bla*_CTX-M-15_, and in the backbone sequence.

Figure 1 shows the genetic structures of CTX-M-15-encoding plasmids isolated from nine ST307 isolates. They shared high similarity in the AMR gene cassette, including *bla*_CTX-M-15_ and *bla*_TEM-1_, and in the backbone sequence. However, some of the plasmids had unique structural features. Compared with the representative plasmid pNR5140, pNR5232 contained a duplicated and inverted AMR gene cassette, whereas pNR5131 contained a fragmented AMR gene cassette (Supplemental Material; Fig. S1). Although these plasmid sequences were compared with the GenBank database using BLASTn, no similar registered sequences from Japan were identified. However, several highly homologous plasmid sequences from other countries were identified (Supplemental Material; Table S2). One of them, pB16KP0226-1, identified in a *K. pneumoniae* ST307 isolate from South Korea (GenBank accession number CP052506.1), is also shown in Fig. 1.

### Amino acid substitutions in the quinolone resistance-determining regions

To investigate the high resistance rate to LVX in ST307 isolates, we analyzed amino acid substitutions in the quinolone resistance-determining regions (QRDRs) of *gyrA* and *parC* in nine isolates subjected to WGS. All nine isolates harbored the GyrA S83I and ParC S80I substitutions.

### Core-SNP analysis

**Fig 2:**
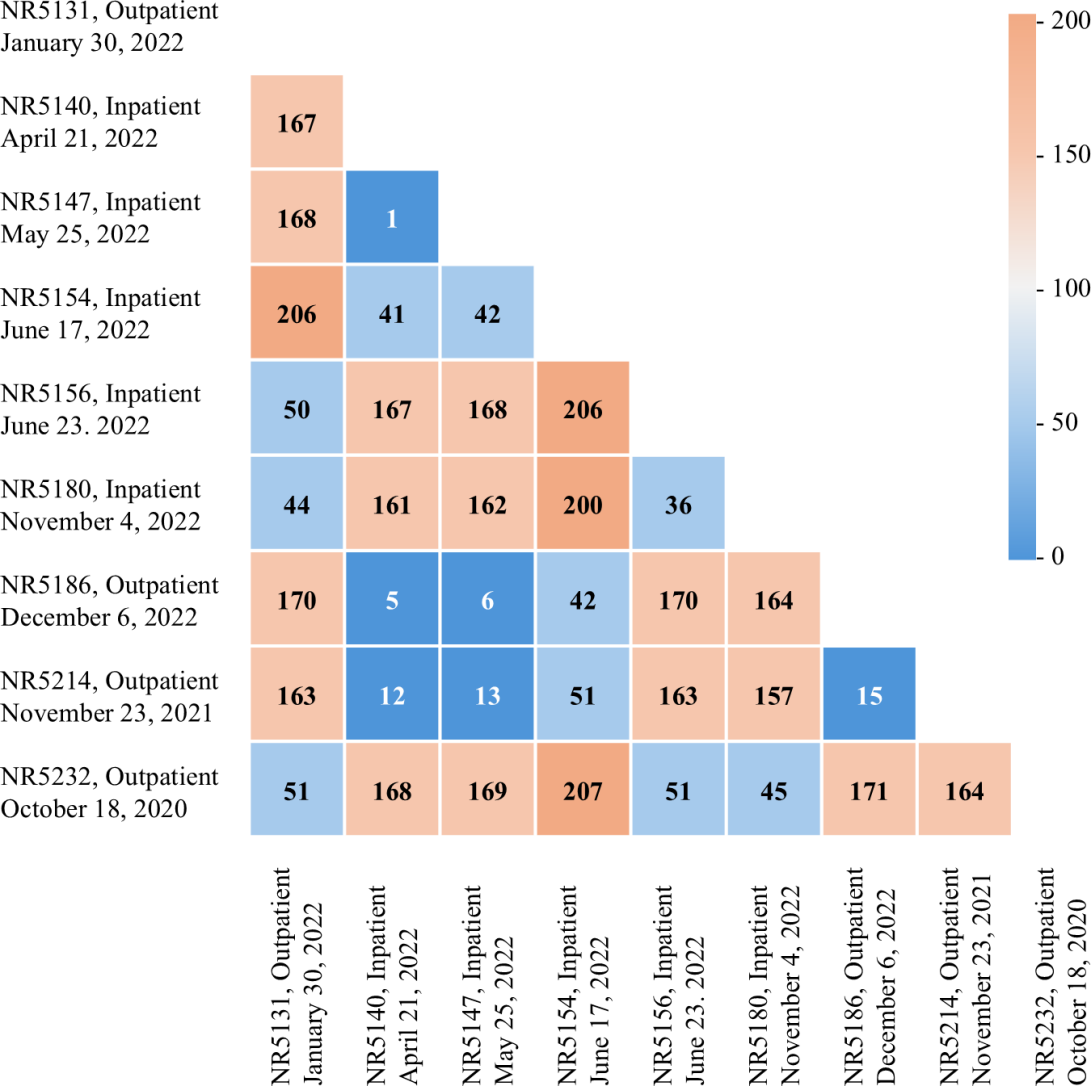
The SNP distance of nine *Klebsiella pneumoniae* ST307 isolates subjected to WGS. Two inpatient isolates (NR5140 and NR5147) and two outpatient isolates (NR5186 and NR5214) revealed a narrow distance, suggesting clonal identity.

To investigate the genetic relatedness of the isolates, core-SNP analysis was performed on nine ST307 isolates subjected to WGS (Fig. 2). The core genomes of the four isolates, NR5140, NR5147, NR5186, and NR5214, differed by 1–15 SNPs, strongly suggesting a clonal identity. These four isolates harbored highly homologous plasmids (Fig. 1). NR5140 and NR5147 were isolated from inpatients in April and May 2022, respectively. However, the patients from whom they were isolated stayed in different wards (data not shown). NR5186 and NR5232 were isolated from outpatients, and their isolation dates were further apart, in November 2021 and October 2020, respectively.

### Profiles of capsular types, virulence genes, and hypermucoviscous phenotype

Among the 119 ESBL-Kp isolates, 3 had the K1 capsular serotype and 19 had the K2 serotype. Of the 22 isolates with K1 or K2 capsular serotype, 3 belonged to an AMR high-risk clone: 2 were K2-ST14 and 1 was K2-ST101. Among the 119 ESBL-Kp isolates, 11 harbored virulence genes. All 11 carried *rmpA* and *iroN*, and 8 also carried *rmpA2* and *iutA*. Of the 11 isolates with virulence genes, 7 belonged to a potentially hypervirulent clone: 1 was K1-ST23, 3 were K2-ST25, 1 was K2-ST65, and 2 were K2-ST86. None of these belonged to an AMR high-risk clone. Among the 119 ESBL-Kp isolates, four exhibited the hypermucoviscous phenotype, of which none belonged to an AMR high-risk clone. None of the ST307 isolates had K1 or K2 capsular serotype, virulence genes, or exhibited the hypermucoviscous phenotype.

## DISCUSSION

No *K. pneumoniae* ST307 isolates were detected in Japan prior to 2014. ST307 was first detected in Japan between 2014 and 2016 in clinical isolates from three regional university hospitals, although the detection rate was low (approximately 2.5%) (5). Recent genomic surveillance has shown a slight increase in the proportion of ST307 isolates in Japan (7). This study is the first to demonstrate rapid regional expansion of ST307 in Japan and suggests that the gradual increase of ST307 over the last decade has accelerated in recent years. In this study, ST307 was isolated not only from inpatients but also from outpatients and newly admitted patients (22 out of 60 isolates), suggesting that the high frequency of ST307 isolation may be due to its dissemination in the community.

TZP is recognized for its potential as a carbapenem-sparing agent for ESBL-producing bacterial infections (28). However, TZP treatment for infection due to Enterobacterales co-harboring ESBL and OXA-1 may lead to worse patient outcomes (28, 29). OXA-1 is a narrow-spectrum oxacillinase not inhibited by tazobactam. In Japan, in 2012, Matsumura et al. (30) reported that only 2.9% of ESBL-producing *E. coli* had OXA-1. However, data on the prevalence of Enterobacterales with OXA-1 in Japan are limited. Because of limited surveillance, Enterobacterales co-harboring ESBL and OXA-1 have been assumed to be rare in Japan, leading some experts to use TZP for treatment. Recently, however, Itadani et al. (31) found that 9.4% of CTX-M-type ESBL-producing *E. coli* in the Chubu region of Japan harbored OXA-1. National genomic surveillance has also shown that 11.2% of ESBL-producing *E. coli* and 17.8% of ESBL-Kp harbor OXA-1 (7). These findings suggest an increase in Enterobacterales carrying ESBL and OXA-1 in Japan. In this study, 53% of ESBL-Kp harbored OXA-1, and compared with non-ST307 isolates, ST307 isolates had a higher prevalence of OXA-1 (88% vs 17%). The spread of ST307 also means an increase in ESBL-Kp co-harboring OXA-1 in Japan. Therefore, careful decisions are needed when considering TZP for ESBL-Kp infections. Furthermore, WGS revealed that *bla*_OXA-1_ is located on the same plasmid as *bla*_CTX-M-15_. Conjugation experiments showed that this plasmid can be easily transferred to *E. coli*. This suggests that CTX-M-15 and OXA-1 may spread together to other bacterial species beyond *K. pneumoniae*, raising concern that treatment options for ESBL-producing Enterobacterales infections could become limited.

Comparison of the ST307 and non-ST307 isolates revealed that ST307 had higher AMR rates, consistent with previous reports from other countries. Among these, the LVX resistance rate was particularly high. ST307 is known to possess characteristic amino acid substitutions within the QRDRs of chromosomal GyrA and ParC, most commonly S83I/D87N in GyrA and S80I in ParC (8). These alterations reduce the binding affinity of fluoroquinolones to DNA gyrase and topoisomerase IV, resulting in high-level resistance to fluoroquinolones. All nine ST307 isolates analyzed in this study exhibited GyrA S83I and ParC S80I substitutions. Similar to ESBL-Kp ST307, *E. coli* ST131 is a well-known AMR high-risk clone that frequently harbors CTX-M-15 and fluoroquinolone resistance (32, 33). The pandemic of *E. coli* ST131 is thought to be driven, at least in part, by selection pressure from fluoroquinolone use (34–36). Likewise, fluoroquinolone-resistant ESBL-Kp ST307 clones may be under similar selection pressure, potentially contributing to their rapid emergence, especially in Japan, where fluoroquinolone overuse has been reported (37, 38).

In this study, two mCIM-positive isolates producing IMP-6 were excluded, and MLST analysis showed that they belonged to ST200 and ST268, not ST307 (Supplemental Material; Table S1). No ST307 isolates carrying both ESBLs and carbapenemases were found. Consistent with the results of our previous study, this study suggests that carbapenem-resistant *K. pneumoniae* ST307 is rare in Japan (6). The results of this study, together with our earlier report, may reflect the extremely low prevalence of carbapenem-resistant and carbapenemase-producing Enterobacterales in Japan (39–41). Although such isolates are uncommon, IMP-type carbapenemases are frequently found among the limited number of carbapenemase-producing Enterobacterales detected in Japan (42, 43). We recently reported the first case of *K. pneumoniae* ST307 carrying IMP-6 globally, which was isolated from a patient in a Japanese hospital (39). ST307 is recognized as an AMR high-risk clone with the potential to carry carbapenemases such as OXA-48, KPC, and NDM-1 (11–13). These findings suggest that the molecular epidemiology of ST307 in Japan may differ from that in other countries. ST307 strains carrying IMP-type carbapenemases may emerge as a unique molecular epidemiological feature in Japan in the future, and ongoing surveillance of their prevalence will be necessary to prevent their spread.

None of the ST307 isolates had capsule types or virulence genes associated with hypervirulence, and they did not show the hypermucoviscous phenotype. To date, ST307 has not shown higher virulence than that of other clones. The relatively low virulence of ST307 may facilitate its dissemination in the community. However, some reports have described ST307 with certain virulence genes or the hypermucoviscous phenotype (44, 45). In Japan, hypervirulent ST307 clones currently appear to be uncommon but have the potential to emerge in the future. This is a cause for concern because it could complicate the treatment of *K. pneumoniae* infections. Attention should be given to the evolutionary dynamics of ST307, particularly its potential to acquire hypervirulent traits.

We compared the genetic structures of plasmids harboring CTX-M-15 in ESBL-Kp ST307 isolates and found high similarity in both the AMR gene cassette, including *bla*_CTX-M-15_ and *bla*_TEM-1_, and the backbone sequence. To our knowledge, plasmid structures of ST307 isolates in Japan have not been reported previously. Structural comparison using BLASTn did not reveal any matches from Japanese isolates. In contrast, several plasmids registered overseas showed high homology, most of which were also harbored by ST307 strains. Given that reports of ST307 increased globally before its detection in Japan, ST307 may have been introduced into Japan from other countries and subsequently started disseminating. Although detailed travel and medical histories were not available in this study, the high homology to non-Japanese plasmids raises the possibility of introduction through international travel, overseas healthcare exposure, or contact with foreign visitors to Japan. Further epidemiological data are required to identify potential importation routes. Among the five plasmids obtained from inpatient isolates, three (NR5140, NR5147, and NR5154) showed high structural similarity, suggesting clonal transmission within the hospital. However, similar plasmid structures were also identified in two outpatient isolates (NR5186 and NR5214) and showed high similarity to plasmids reported in isolates from other countries. These findings suggest the possibility that ST307 strains carrying this plasmid may have a high potential for dissemination. Furthermore, several plasmids showing high homology to the representative plasmid in this study were found in non-ST307 strains, including ST11 and ST405 (Supplemental Material; Table S2). This finding suggests that the plasmid has the potential to spread across different STs and may also form a broader epidemiological reservoir extending beyond nosocomial settings, although comprehensive plasmid analysis of non-ST307 lineages is required to confirm this hypothesis. The plasmid structure identified in this study may serve as an epidemiological marker for both ST307 and other AMR high-risk clones, particularly in Japan, where such high-risk elements remain uncommon. These observations highlight the need for ongoing surveillance.

The plasmid characteristics revealed by WGS suggest the potential for both nosocomial transmission and community dissemination of ST307. This finding is further supported by core-SNP analysis. The core-SNP analysis indicated clonal identity between NR5140 and NR5147. Although they were obtained from patients in different wards, their isolation dates were 1 month apart, raising the possibility of nosocomial transmission via contaminated hands of healthcare workers, shared equipment, or the hospital environment. Although contact precautions, including gown and glove use and dedicated patient equipment, were implemented for all patients colonized or infected with ESBL-Kp, the possibility of silent transmission cannot be excluded because routine screening of contacts was not performed. In addition, the analysis revealed a narrow SNP distance between the outpatient (NR5186 and NR5214) and inpatient (NR5140 and NR5147) isolates, despite the isolation dates of the outpatient isolates being widely separated in time. These findings suggest that the same ST307 clone may be spreading in the community.

Our findings support both national and international recommendations emphasizing antimicrobial stewardship and infection control. Japan’s National Action Plan on AMR (2023–2027) (46) and WHO guidelines for the prevention and control of carbapenem-resistant Enterobacteriaceae, *Acinetobacter baumannii*, and *Pseudomonas aeruginosa* in healthcare facilities (47) stress the importance of surveillance and prevention strategies for multidrug-resistant organisms. In this context, the increasing prevalence of ST307 harboring CTX-M-15, OXA-1, and high-level resistance to LVX highlights the need for strengthened stewardship programs and genomic surveillance in both hospital and community settings.

This study has three main limitations. First, it is a single-center study. Therefore, the findings primarily reflect the situation of this hospital and its local community rather than nationwide trends in Japan. In addition, although ST307 isolates were detected in almost all wards, eight cases clustered in one ward in 2022, suggesting possible nosocomial transmission. Nevertheless, given the small number of clustered cases, nosocomial transmission is unlikely to have had an important effect on the overall results. Multicenter studies incorporating more detailed temporal and ward analyses, together with use of molecular typing methods such as pulsed-field gel electrophoresis or core genome MLST, are warranted to clarify transmission patterns. Second, the genetic analysis was limited. MALDI-TOF MS may not reliably distinguish *K. pneumoniae* from closely related species, and PCR-based detection targeting selected genes may have overlooked rare β-lactamases or additional virulence determinants (48). Furthermore, WGS was performed on only nine of the ST307 isolates and none of the non-ST307 isolates. Broader WGS using analytical tools such as Kleborate (a tool to screen genome assemblies of *Klebsiella pneumoniae* and the *Klebsiella pneumoniae* species complex) is required to improve species identification and evaluate genetic diversity, plasmid evolution, and the potential acquisition of AMR and hypervirulence of both ST307 and non-ST307 isolates. Third, both causative pathogens and colonizing bacteria were included, but the clinical impact of ST307 dissemination was not assessed. Although colonizing isolates were intentionally included to better capture the transmission dynamics, this approach may have introduced bias regarding clinical severity or population representativeness. Given the microbiological features of ST307, patients with prior fluoroquinolone exposure may be at higher risk of ST307 carriage, and the use of TZP for ST307 infections may lead to poorer outcomes. Further evaluation of the clinical features and infection control strategies for ST307 infections is warranted. We are planning to conduct a clinical study focusing on ST307 infections.

In summary, this study is the first to report the high prevalence of ESBL-Kp ST307 isolates in Japan. Our findings suggest a rapid spread of ST307 in our region. Ongoing surveillance is warranted to monitor the dynamics of these AMR high-risk clones, including changes in the associated plasmids and resistance genes.

## Data Availability

The genome sequencing data have been deposited in the DDBJ database under BioProject accession number PRJDB35476. The corresponding BioSample accession numbers are SAMD01184753–SAMD01184761, which include *Klebsiella pneumoniae* isolates NR5131, NR5140, NR5147, NR5154, NR5156, NR5180, NR5186, NR5214, and NR5232. All data are publicly available at DDBJ (https://www.ddbj.nig.ac.jp/index-e.html).

## SUPPLEMENTAL MATERIAL

Fig. S1 spectrum.02526-25-s0001.pdf The genetic structures of two characteristic plasmids.

Supplemental tables spectrum.02526-25-s0002.xlsx Tables S1 and S2.

ASM does not own the copyrights to Supplemental Material that may be linked to, or accessed through, an article. The authors have granted ASM a non-exclusive, world-wide license to publish the Supplemental Material files. Please contact the corresponding author directly for reuse.
