# Supplementary material for: Emergence and regional spread of extended-spectrum β-lactamase-producing Klebsiella pneumoniae ST307 at a Japanese tertiary-care hospital
Source: Microbiol Spectr. 2025 Dec 12;14(2):e02526-25. doi: 10.1128/spectrum.02526-25 (PMC12889025; doi:10.1128/spectrum.02526-25)
Supplement: Fig. S1 — The genetic structures of two characteristic plasmids. [file spectrum.02526-25-s0001.pdf]

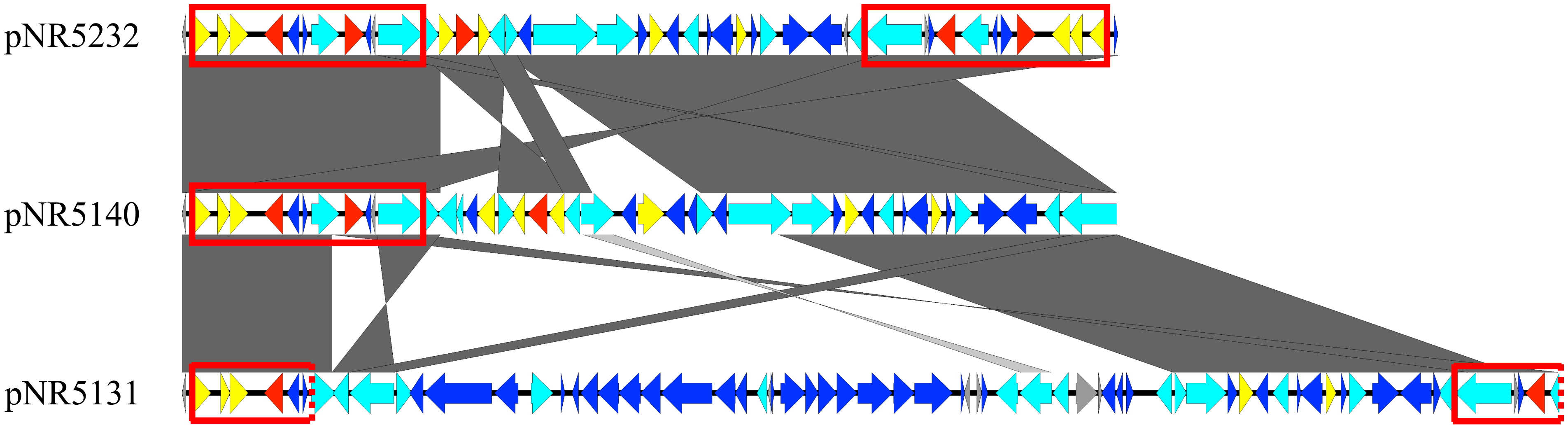

FIG S1 The genetic structures of two characteristic plasmids, pNR5232 (top) and pNR5131 (bottom), compared with a representative plasmid, pNR5140 (middle). pNR5232 contains a duplicated and inverted AMR gene cassette, and pNR5131 has a fragmented AMR gene cassette. Abbreviations: AMR, antimicrobial resistance.
